# The effect of the Educational Scholar Program as a longitudinal faculty development program on the capability of educators as scholars

**DOI:** 10.1186/s12909-023-04682-7

**Published:** 2023-09-22

**Authors:** Fatemeh Keshmiri

**Affiliations:** 1 Medical Education Department, Education Development Center, Shahid Sadoughi University of Medical Sciences, Yazd, Iran; 2 National Agency for Strategic Research in Medical Education, Tehran, Iran

**Keywords:** Educational Scholarship, Scholarship of teaching and learning, SoTL, Faculty Development, Longitudinal program, Teaching Scholar Program, Educational Scholar Program, Kirkpatrick model, Learner-related outcomes

## Abstract

**Introduction:**

The Educational Scholar Program (ESP) is designed and implemented as a longitudinal and institution-based faculty development program. The present study aimed to assess the effect of the ESP on educators’ capabilities to undertake SoTL activities associated with their scholar role.

**Methods:**

This study was conducted from 2017 to 2022. The participants (n = 64) were educators in six schools of Shahid Sadoughi University of Medical Sciences. The ESP was a faculty development program that consisted of training and project-based stages. The educators experienced small-group learning, self-directed learning, and reflective assignments in the training stage. In the second stage, the educators completed a SoTL (Scholarship of Teaching and Learning) project. Learner-related outcomes based on Kirkpatrick model was assessed. The reaction of educators (satisfaction, active participation in the ESP, and the perception of mentoring sessions) was assessed by three questionnaires (Reaction level). The educators’ learning was evaluated by modified essay questions and their project reports (Learning and Behavior levels). Outputs of the ESP including journal publications, abstracts presented at meetings or congresses, grant funding, awards in educational festivals, promotions, projects with ongoing implementation following the ESP, and conducting further SoTL projects after ESP were assessed quantitatively over two years after participating in the ESP (Results level). Data were summarized by descriptive statistics (mean, percentage, SD, 95% Confidence Interval (CI)). Cut-off scores of the instruments was calculated with a standard setting method which introduced by Cohen-Schotanus and Van DerVleuten. Data analyzed by One-sample t-test.

**Results:**

Sixty-four of 72 (89%) educators completed the ESP. The mean (CI) satisfaction score of educators was 42 (CI: 26.92–58.28), the active participation was 92 (CI: 80.24-103.76). The scores of the mentoring assessment from the perspective of the educators were reported at 90 (CI: 78.24- 101.76). The mean (95%CI) learning scores in the essay examination were 88 (CI: 70.36- 105.64), and project assessment were 90 (CI: 78.24- 101.76). The results showed the educators’ scores in reaction and learning significantly higher than the cut-off scores. (P < 0.05). Most projects were conducted in curriculum development and assessment/evaluation domains. The number of projects with ongoing implementation over the two years following the ESP and the acquisition of grants was higher than other outputs in the results level.

**Conclusion:**

The ESP, as an institute-based longitudinal program, enhanced the learner-related outcomes (in four levels of reaction, learning, behavior, and results). The creation of practical learning and supportive mechanisms influenced on the results. The outcomes of ESP indicated that the educators prepared to conduct SoTL activities in their educational community.

**Supplementary Information:**

The online version contains supplementary material available at 10.1186/s12909-023-04682-7.

## Introduction

Faculty development programs (FDPs) aim to improve education effectiveness according to the faculties’ needs and the organization’s policies [1]. FDPs provide situations for fostering the faculties’ capabilities to be compatible with changing environments in educational systems and emerging advanced educational strategies [1–4]. FDPs facilitate the professional development of faculty members and the personal growth of those who need to pursue a career as a medical educator or educational leader [5, 6].

On the other hand, a significant responsibility of faculty members is to participate in educational scholarship and disseminate their scholarly work to scientific and professional communities [5]. This responsibility helps faculty members align with educational changes, adapt to technological and methodological innovations, and excel in teaching [7–12]. Hence, the Educational Scholar Program (ESP) or Teaching Scholar Program (TSP) are recommended to improve the faculties’ capabilities in SoTL [3, 13]. In ESP, educators experienced scholarly work in teaching-learning, curriculum development and evaluation, educational leadership, and learning theories [14, 15]. Chandran and colleagues [3] designed a National Longitudinal Faculty Development Curriculum focused on educational scholarship. This program enhanced educators’ academic knowledge and skills and created a professional network. Macario developed a Stanford Anesthesia Faculty Teaching Scholars Program to improve faculties’ scholarly preparation. The ESP has benefited faculty development and advanced educational improvements in the Department of Anesthesiology [16]. Muller and Irby also used ESP to train educational leaders at the University of California and San Francisco [17]. Keshmiri, in a qualitative study, explored ESP as a developmental pathway toward leadership from the perspective of educators [12].

Steinert et al. reviewed 13 ESPs as longitudinal programs in a BEME guide. They showed that only a few interventions led to educational excellence and scholarship enhancement in the organizations, which remains a key area for further studies [1]. In this regard, Moses et al. showed that ESP had a small effect on productivity and faculty retention [18]. In another study, Steinert acknowledged that additional studies are required to clarify various dimensions of ESP in the educational community [14].

The present study implemented ESP at Shahid Sadoughi University of Medical Sciences (SSU) in Iran and tried to answer the following question: ‘What was the effect of ESP on the learner-related outcomes (reaction, learning, behavior, and result levels)?‘

## Methods

This study was conducted from 2017 to 2022.

### Study setting

As an institute-based program, the ESP was conducted at SSU in 2017. The university includes six schools: medicine, nursing and midwifery, public health, paramedical sciences, pharmacy, and dentistry. All schools were represented in the ESP cohorts.

The Education Development Center (EDC) in the university comprises a committee for scholarship support in the educational field (projects of ‘research in education’ and SoTL). The committee’s annual budget for financial support and grants at the institution level was 30 million IRR from 2011 to 2016. Six grants and two publications were reported from 2011 to 2016. The budget of six projects was 140 million IRR.

The developmental policies of educational scholarship in medical sciences education have been implemented in Iran by the Ministry of Health and Medical Education and the universities of medical sciences. Thus, educational scholarship activities were supported in the universities by forming SoTL committees in the EDC, developing FDPs to foster a culture of SoTL in the universities, and defining educational scholarship activities to promote educators. Financial support and grants for SoTL activities were also planned at the institute (internal grants awarded for SoTL projects) and the National Agency for Strategic Research (NASR). The annual budget of the SoTL committee of the institute was 300 million IRR ~ 600 USD (1 USD ~ 500,000 IRR, on February 20th, 2023). The acceptance rate was 80% in the ‘SoTL committee’ and 60% in the ‘Research in Education committee’ of the institute. The total SoTL grant pool of NASR for 170 SoTL projects was about 12,000 million IRR in 2019–2022.

### Participants

Participants: A total of 72 educators who participated in the ESP program were identified as eligible participants for this study through a census, and all of them willingly agreed to take part in the research.

Facilitators and mentors: They were faculty members of health profession education (n = 2), psychology (n = 2), and psychiatry (n = 2). They have working experience in SoTL activities for 8 years ± 1.5, faculty development programs for 10 years ± 3, and mentoring programs for 3 years ± 1. The same individuals were facilitators in stage 1 (training) and mentors in stage 2 (project-based learning). The mentors to facilitators ratio is 2.3.

The facilitators and mentors attended the sessions for orientation and review of the ESP ground rules. These meetings were held in four face-to-face sessions for eight hours. The sessions aimed to help facilitators and mentors become familiar with key activities in each stage, their roles as ‘facilitators’ in the training stage, and ‘mentors’ in the project-based stage. Compliance with the principles of adult learning and using interactive methods were emphasized in implementing the ESP. They also discussed the teaching-learning methods, educational content, project-based learning process, and ESP’s formative and summative evaluation methods.

Moreover, they reviewed the assessment tools and debated how to conduct the assessment process; they also discussed their scoring system and how to provide feedback. The communication channels (including WhatsApp) and meeting schedules were determined to share their experiences and issues during the ESP. An executive manager of the ESP directed the sessions.

This study devoted much time to scheduling briefing meetings for the facilitators and mentors. They participated in all cohorts of ESP and directed SoTL projects in the educational community. To ensure the sustainability of mentors and facilitators, they were involved in the different committees of SoTL in the EDC in the institute and the schools.

The ESP was directed and supervised by an executive manager with a Ph.D. in Medical Education and seven years of experience in SoTL activities (the author).

### Theoretical framework

The ESP was developed using the project-based learning approach as a theoretical framework. The project-based learning included beginning inquiry (asking questions, formulating goals, planning procedures, and designing investigations), directing inquiry (conducting data searches, constructing methods, and collecting data), analysis and critical reflection (analyzing data, drawing conclusions, collaborating on written work), and disseminating knowledge and seeking feedback (giving and seeking feedback) [19, 20]. In the ESP, project-based learning was consistent with the model of SoTL introduced by Rowland and colleagues [21]. The steps were organized into three parts: (1) idea generation and study design, (2) implementation, (3) analysis results, critical reflection, and dissemination [21].

### A summary of the educational scholar program

The ESP was designed as a longitudinal and institution-based faculty development program. Institution-based ESP provides opportunities to develop networks of peers and collaborators aimed to share their experiences of problem-solving processes and social support activities. Longitudinal ESP allows educators to experience the process of SoTL from the designing stage to dissemination [3, 22].

The educators who had at least nine months of teaching experience in different fields and had completed the faculty development program of ‘basic principles of education’ were admitted to the ESP. Since the ESP, as a longitudinal FDP, lasted about 18 to 23 months, educators with less than 23 months of tenure at the university were excluded from the program during the registration process (Because they left the university before completing the ESP course).

The educators voluntarily enrolled in the ESP, and about 14 enrolled in each cohort.

### Main educational principles in ESP

The main objective of the ESP was to prepare educators to undertake educational roles and related scholarly activities. The educational role of a scholar requires choosing the best practice in educational activities by using informed decision-making and improving the quality of the educational process [23].

The educational principles of the ESP:

The ESP Adult learning principles process included the learner’s motivation, problem-centeredness, relevancy-oriented, goal-oriented, and self-directed [24]. The principles were applied in two stages of the ESP, i.e., connecting professional and career development, individualizing learning, engaging in various sources of learning, supporting professional development, ensuring continuing professional development, encouraging mentoring, providing data-informed and job-embedded learning, and planning for professional development [24].

Collaborative learning was also implemented in the ESP by participants’ contributions to learning activities [25]. Social interaction, individual accountability, and the development of shared practices were key factors in the collaborative learning activities [26–28]. These factors were considered in two stages: training and project-based learning.

Interactive learning methods were used in different learning situations, such as small group settings, problem-oriented methods, self-directed learning in the training stage, and project-based learning, mentoring, and collaboration networks in the second stage [21]. The interactive teaching-learning methods in the training stage provided a situation for discussing and practicing new skills and reflecting on their learned knowledge [1, 29–31].

The formative and summative evaluation of the ESP process and learner-related outcome was conducted based on the Kirkpatrick model [32–35]. A formative process evaluation of the ESP as a dynamic program [33] was conducted within each cohort by the executive manager of the ESP. The SoTL criteria, including clear goals, appropriate methodology, adequate preparation, effective presentation, significant results, and reflective critique of their projects [36] was used to assess SoTL projects.

#### Mentoring in ESP

Individual mentoring was done in the ESP. Each mentor directed 2–3 educators in each cohort. Regular meetings of mentors and educators were planned. The mentor and educators described ground rules at the beginning of the mentoring session. They discussed the expectations, duties, priorities, relationship channels, regular meeting times, and commitment to confidentiality.

Moreover, the objectives and priorities concerning personal and professional objectives, the plan for the learning opportunities, the executive process of the SoTL project, evaluation methods, and financial affairs were argued. The mentor guided the educators in the design and development process of a SoTL project. The mentor supported the educators by introducing resources to guide self-directed learning, providing feedback, facilitating the learning process, and implementing the SoTL project. The educator reported on the progress of the SoTL project to the mentor in regular meetings. During this stage, the mentoring meetings were scheduled for at least two monthly sessions, face-to-face and online. These sessions were about forty, and each took 40–60 min. Each educator participated in about 35–40 mentoring sessions.

#### Collaboration network

The collaboration network was established in ESP to facilitate educators’ relationships with mentors and peers and share their experiences. The network provided opportunities for the educators to present their experience about the design and implementation of the SoTL project. They also discussed the perceived challenges and solutions in the network and used the critical opinions of others (educators and mentors) to improve their projects.

The participants’ interactions were facilitated by face-to-face and online group discussions and virtual groups on WhatsApp social networks. All educators actively participated in these meetings and shared their SoTL project at least twice during their ESP cohort. All mentors and educators in each cohort participated in the collaboration network, which was scheduled monthly.

#### ESP stages

The ESP consisted of the training (5 months) and the project-based stages (13–17 months) (Table 1).

**Table 1:** Overview of ESP Training Stage

	Topic	Objectives	Participants’ Activities	Number of teaching sessions
Step 1	Education Theory and Models	- Describe theories and modes in different domains of education	- Interactive discussion about the content and their applications	6
Step 2	Study Design in SoTL activities	- Reviewing papers with different methodologies in education - Critically analyze SoTL activities using different study designs	- Analyze topic-specific journal articles - Discuss the benefits and challenges of different study designs - Discussion of a proper design and methodology in different SoTL cases	4
	Scholarly Dissemination	- Recognition of potential channels for dissemination of SoTL	- Search a listing of educational conferences, journals, or other sources that accept SoTL - Find a topic-specific journal for their idea.	2
Step 3	Critical appraisal and Peer Review of SoTL projects	- Describe of review criteria of SoTL - Critically analyze SoTL - Peer review of SoTL	- Discuss the critical criteria of a peer review - Peer review a scholarly paper or project - Analyze topic-specific journal articles	4
Step 4	Formulation a SoTL proposal in a small groups	- Apply the theories to their practice and the SoTL project	- A search of the literature on the topic - Analyze topic-specific articles	4

Training stage: This stage consisted of 20 sessions in a weekly schedule for five months, and each session lasted two hours. The stage consisted of four steps: (1) learning about education theory and models, (2) learning about study design in SoTL activity and scholarly dissemination, (3) critical appraisal and peer review of SoTL projects, and (4) formulating a SoTL proposal in small groups. The main teaching-learning methods included interactive lectures, small group discussions, flipped classrooms, JIGSAW (Appendix 1) [37]., and case-based learning [38].

Step 1–1: The educators reviewed educational theories, models, and evidence-based education in the following domains: curriculum development (goal setting, need assessment, advanced approaches), teaching-learning methods (the interactive and learner-centered methods and problem-solving methods), and evaluation (student assessment methods, and curriculum evaluation models). In addition, the educators learned about the SoTL concept, steps, and evaluation criteria. In this step, the educators were asked to write reflective assignments focused on theory and academic evidence. The reflective assignments aimed to compare and establish the relationship of theory and academic evidence to the educators’ practice and interpret their experiences. The expected word count for the assignments was 2,000 words.

Step 1–2: The educators were divided into small groups (3–5 educators) and participated in group activities. The purpose of these activities was to create opportunities for educators to learn from each other and together. They experienced different roles in educational scholarship activities, such as team leader, course developer, lecturer, facilitator, and moderator. Educators learned how to design SoTL projects through a systematic and evidence-based approach in this step. They learned it by reviewing the research methodology in their field of study and SoTL projects in small groups.

Step 1–3: The educators contributed to journal club sessions to critically appraise educational articles regarding methodology and content. The educational theory/models, teaching-learning, and evaluation methods of SoTL were critically appraised in the sessions. In addition, the educators analyzed SoTL projects (which have received awards in a national educational scholarship festival called the Motahary Festival) in small groups.

Step 1–4: The group activities were designed in this step. The educators practiced the formulation of the SoTL proposal in their small groups. Peers reviewed the proposals by a 10-item checklist [39] (Explained in the ‘Measures’ section).

Project-based stage: In this stage, the educators were asked to develop and implement a SoTL project. This stage was organized into three steps: (1) idea generation, study design, and getting prepared for implementation; (2) implementation and examination of in-progress results; and (3) analysis of results, dissemination, and critical reflection [21].

To this end, the educators were asked to assess their educational needs to solve educational problems and improve the quality of education. Then, they were asked to find solutions for improving the quality of their education by consulting with peers and reviewing the literature. Subsequently, the educators were asked to formulate a SoTL proposal for their idea under the supervision of their mentor. Their proposal was peer-reviewed by a SoTL committee (consisting of 12 experts in health professions education (n = 3) and clinical teachers (n = 9) who had at least five years of experience in SoTL activities at the Education Development Center of the university. After that, the projects were applied to obtain a grant.

To implement SoTL, the educators prepared the requirements for conducting their projects in the educational environment. The SoTL projects were implemented in the educational community. All steps of the design, implementation, evaluation, and management of a SoTL were guided by a mentor. The educators presented their SoTL report. The expected word count for SoTL project reports was 3000–5000 words. Finally, the SoTL committee evaluated the SoTL reports by a checklist according to the six criteria of SoTL.

### Formative and summative evaluation

The evaluation of the ESP process and learner-related outcomes of the ESP was conducted based on the Kirkpatrick Model [34]. The executive manager of the ESP supervised formative and summative evaluations of the ESP.

### Formative and summative assessment of educators

The formative evaluation of the educators’ learning was conducted by peer-reviewing the SoTL project in designing, implementing, and disseminating stages. The educators received regular feedback in their network and small group meetings. The mentors evaluated the educators in their group. In addition, the number of individual mentoring sessions and the active participation of educators in the collaboration network were monitored, and feedback was provided to the educators and the mentors by the executive manager of the ESP.

#### Summative evaluation of educators

The educators’ reactions, learning, and behavior were evaluated using the Kirkpatrick model (Described in the ‘Measures’ section below).

#### Formative and summative evaluation of ESP

Formative process evaluation of the ESP: The main questions of process evaluation were ‘How was the program implemented, compared to the initial plan?‘ and ‘Was the program running efficiently? If not, why?‘ [33]. The evaluation was conducted by reviewing documentation of ESP sessions and assessing the viewpoints of the educators and mentors through periodic telephone calls (response rate = %67, (n = 43)) and electronic contact (response rate = %78 (n = 50)) and face-to-face meetings (response rate = %70 (n = 45)) ongoing within each cohort by the executive manager of the ESP. The responses were classified by a directed content analysis method [40] into two categories: program implementation (e.g., teaching-learning process, content coverage, and active participation of educators and mentors) and program improvement (e.g., suggestions of effective learning for knowledge application). The feedback was discussed in panels of directors, mentors, and facilitators and was planned to continue the improvement of the ESP.

Summative evaluation of ESP: The success of an SoTL project is measured through criteria introduced by Glassick [41]. (Behavior and Result levels of the Kirkpatrick model). The ‘successful completion’ of SoTL projects was achieved when the projects fulfilled six of Glassick’s criteria. Besides, SoTL profiles of educators were assessed quantitatively over time (Result level) (As described in the ‘Measures’ section below).

**Passing the ESP standard:** A passing standard was derived from active participation in 80% of activities and meetings and achieving a cut-off score in evaluating participants’ learning in the essay and project assessment.

Educators who passed the ESP received awards such as professional development and SoTL points in their teaching portfolio. These achievements are considered in educators’ promotion and tenure process.

### Measures

**Reaction level:** Three questionnaires assessed the reaction of the educators: (1) satisfaction, (2) active participation of educators, and (3) the perception of mentoring.

1) A 9-item questionnaire assessed educators’ satisfaction with the ESP. The questionnaire was developed and validated in a previous study (Cronbach’s alpha = 0.91) [42]. The questionnaire was completed by self-report. The scoring was from 1 (strongly disagree) to 5 (strongly agree). The minimum and maximum scores were 9 and 45, respectively.

2) A 10-item instrument evaluated the educators’ active participation in the ESP. This instrument was developed based on the literature review and expert opinion. The face and content validity of the instrument was approved from the viewpoints of 20 experts in health profession education. Correspondingly, the internal consistency of the instrument was approved (Cronbach’s Alpha = 0.87). The active participation of educators in mentoring sessions and the collaboration network were evaluated by their mentors and the executive manager of the ESP (the author). The items were scored in the range of 1 to 10. The minimum and maximum scores of the instrument were 10 and 100, respectively.

3) The educators assessed the mentoring in ESP through a 10-item instrument at the end of the cohort. This questionnaire was developed in Zarrabi et al.‘s study [43]. The face and content validity of the questionnaire to be used in the ESP was assessed by 12 experts in health professions education in the present study. According to the experts’ suggestions, two items were removed from the questionnaire. The internal consistency of the questionnaire was approved by Cronbach’s Alpha = 0.89. The items were scored in the range of 1 to 10, while the minimum and maximum scores of the instrument were 10 and 100, respectively.

**Learning level**: The educators’ learning was assessed by modified essay questions. In each cohort, the facilitators developed the modified essay questions according to a blueprint of examination. Twenty questions were developed in different domains of curriculum development, evaluation, teaching and learning methods, and class management. The content and face validity of the questions were confirmed by experts in health professions education (n = 6). Four exam sessions (5 questions in each) were planned in this university’s learning management system (LMS). The time spent on each question was 7–10 min (about 50 min in each exam). The examination was an open book and not invigilated, and it was administrated through a specialized software platform (Navid) that set the exam time. A specific date was set for the exam to be active on the platform. The scoring range was 1 (minimum score) to 100 (maximum score). Scoring of the examination was conducted by an essay grading rubric consisting of four parts: explaining basic concepts/principles, the accuracy of content, organizing, and style. The validity of the rubric was confirmed by the viewpoints of eight experts in health professions education. Two raters (the facilitator and the executive manager) scored the results according to the scoring rubric. The agreement of the raters was ICC = 0.80.

**Learning and behavior levels:** The SoTL projects were evaluated by a 10-item checklist [39]. According to Ahmari et al. study [39], the checklist was developed and validated by 12 experts with at least five years of experience in SoTL activities. The agreement of the raters was ICC = 0.0.86. The scoring range was 1 (minimum score) to 100 (maximum score). In addition, the rate of educators who successfully conducted their SoTL projects after participating in the ESP was assessed.

**Result level:** Outputs of the ESP, including journal publications, abstracts presented at meetings or congresses, grant funding, awards in educational festivals, promotions, projects with ongoing implementation following the ESP, and conducting further SoTL projects after ESP were assessed quantitatively over two years after participating in the ESP.

### Data analysis

Data were summarized by descriptive statistics (frequency, mean, standard deviation, and 95% Confidence Interval (CI)). Inter-rater reliability was assessed by the agreement between two raters (ICC coefficient). Then, the one-sample t-test was used to compare educators’ scores with cut-off scores as a reference value. A cut-off score of the instruments was calculated with a standard setting method introduced by Cohen-Schotanus and Van DerVleuten (the 95th percentile point of the score’s instrument × 60%) [44]. The cut-off score of the instruments was calculated so that active participation = 60, essay examination = 57, project assessment = 56.4, mentoring assessment = 55.8, and satisfaction = 27. The measure change rate (the number of grants, their budgets, and the number of publications) from 2017 to 2022 compared to those that occurred from 2011 to 2016 was calculated by the ‘change percentage’ formula (new value-old value)/old value ×100).

## Results

In this ESP, 72 educators enrolled in five cohorts, and 64 completed the program (89%). The results reported a 4% dropout rate (three educators left the ESP in the training stage) and a 7% rate of failing to complete the ESP (five educators left the ESP in the project-based stage in all cohorts). The educators were asked about the reasons for failing and not continuing the ESP, including the end of employment in this university, transfer to another university, acceptance of new executive responsibility, and lack of time due to new school activities. This study involved 34 females (53%) and 30 males (47%). The participants included 38 assistant professors (60%) and 26 associate professors (40%); their mean age (± SD) was 38 ± 3 years, and their working experience was 6 ± 3 years.

### Formative evaluation findings

Most of the extracted codes in a ‘program implementation’ category were used to explain the following items: matching educational content with the program, using various teaching-learning methods to increase practical learning, using interactive methods and projects/assignments, and focusing on reflection on theory and academic evidence. In the ‘program improvement’ category, the suggestions of mentors and educators were explained using simulation situations for practicing the methods, creating the opportunity to review SoTL reports in various domains, consulting peers and mentors at any time and place, and monitoring mentors and educators. Some suggestions were executed to improve the ESP cohorts, namely access to educational content in the learning management system, using flipped classrooms in more sessions, regularly monitoring the participation of educators and mentors in the collaboration network, and providing feedback.

### Learner-related outcomes

The results did not vary over cohorts and were pooled.

Reaction level: The mean (95% CI) satisfaction score of educators was reported at 42 (CI: 41.2–42.7, P = 0–0001). The mean (CI) scores of active participation of educators were reported at 92 (CI: 90.9–93.0, P = 0.0001). The mean (CI) scores of the mentoring assessment from the perspective of the educators were reported at 90 (CI: 89.5–90.3, P = 0.0001) (Table 2).

**Table 2:** The Reaction of Educators in the ESP

Satisfaction scores to the ESP	Mean ± SD
1. Educational objectives were clearly explained.	41.9 ± 11
2. The educational contents and their difficulty level were appropriate.	39.8 ± 10
3. The sequence of contents was appropriate.	40.3 ± 6
4. There was enough opportunity for group discussion and collaborative activities.	44.2 ± 7
5. There were opportunity to reflect on my experiences in training sessions and assignments.	43.9 ± 9
6. There were opportunities to exchange educational experiences with members of different disciplines in a small groups.	43.9 ± 8
7. The teacher/s had the necessary mastery and skills to teaching and manage the training session.	41.9 ± 9
8. I achieved the goals specified at the beginning of the ESP.	43.8 ± 5
9. I can apply what I have learned in the ESP sessions.	43.8 ± 7
**Active participation of educators in the ESP**	
1. Actively participates in group activities.	94 ± 6.6
2. Effective interaction with group members.	95 ± 8
3. Actively collaborate for learning together in interprofessional groups.	93 ± 7.6
4. Performs his/her duties appropriately in group activities.	94 ± 6
5. Creatively develops ideas to solve educational issues in group activities.	88 ± 5.5
6. Contributes appropriately to reflective assignments.	94 ± 6.5
7. Gives feedback to other team members.	88 ± 6
8. Accepts feedback from team members.	89 ± 4.5
9. Shares his/her experiences and learnings with others.	95 ± 6
10. Attends meetings as scheduled.	94 ± 7.7
**The educator perceptions about quality of mentoring**	
1. Creating motivation to participate in SoTL activities	91 ± 8
2. Help to improve personal and professional capability in education	88 ± 7
3. Effective and encouraging communication	93 ± 4
4. Acting as a SoTL facilitator	94 ± 3
5. Deliver constructive feedback	90 ± 5
6. Effective and useful participation of the mentor	90 ± 8
7. Assisting in personal growth by helping to identify strengths and weaknesses	85 ± 9
8. Satisfaction with the mentor	92 ± 4
9. Willingness to participate in mentoring sessions in the future	93 ± 5
10. Follow the schedule by the mentor	90 ± 8

Learning level: The mean (95% CI) educators’ learning scores in the essay examination were 88 (CI: 87.1–88.9), P-value = 0.0001.

Learning and behavior level: The mean (95% CI) educators’ scores of project assessment were 90 (CI: 89.4–90.5), P-value = 0.0001 (Table 3). The most common domains of the SoTL project were curriculum development and assessment/evaluation (Table 4).

**Table 3:** The SoTL Project Assessment

Items	Mean ± SD
1) The title of the project is appropriate.	92 ± 4
2) Literature review contains the necessary and update information.	89 ± 8
3) The goal and objectives are written appropriately.	93 ± 4
4) The study design is suitable for achieving the goals of the SoTL and supported by best evidence.	91 ± 8
5) The method of implementing is in accordance with the desired goals and methodology and are stated in detail.	89 ± 7
6) The ethical considerations of the SoTL are correctly explained.	88 ± 7
7) The results (analyze, present and interpret) in accordance with the desired goals and methodology and are stated in detail.	91 ± 5
8) The critical appraisal, and reflecting on the SoTL project are correctly explained and contains the necessary details.	89 ± 5
9) The necessary details of SoTL project are documented.	88 ± 6
10) The disseminating of the SoTL are correctly explained.	90 ± 6

**Table 4:** The SoTL Subjects in Different Domains

Domains	Funded in SoTL committee in the institute	Funded by the National Grant	Sample of SoTL subjects
Curriculum development	12	9	- Development of the interprofessional collaboration competency framework in the occupational health team - Design, implementation, and evaluation of the short-term empowerment course about educational skills in the medical science system - Designing and implementing an interprofessional education program about interprofessional professionalism in surgical wards
Teaching and learning	9	8	- Designing and implementing an educational program based on Reflection Model among clinical students - Designing an educational program related to the teaching of pharmacology by the ‘concept map’ method - Using a scenario-based method in the teaching of moral reasoning among faculty members’
Educational material	4	5	- Designing and developing of ‘Co-Surgery’ as an educational application for surgical technology students - Development of educational applications regarding professionalism and ethics in education
Evaluation and assessment	4	11	- Evaluation of education development offices in faculties and teaching hospitals - Evaluation of scaling and suturing skills of periodontics residents by using OSATS - Designing and implementing performance evaluation of faculty members by multi-source feedback method - Designing and implementation of clinical reasoning examination in dental school - Designing and implementing performance evaluation of educational managers from the point of view of different stakeholders
Advising/mentoring	1	1	- Design and implementation of student mentoring program in pharmacy school
Total	30	34	

Result level: The outputs of the ESP are shown in Table 5.

**Table 5:** Outputs of the ESP during five cohorts (Result Level of Kirkpatrick model)

Publication in a journal N (%)	22 (34)	Journals: BMC Medical Education (n = 1), BMC Nursing (n = 1), Strides in Development of Medical Education (n = 3), Journal of Education and Health Promotion (n = 2), Journal of Medical Education and Development (n = 7), Development Strategies in Medical Education (n = 1), International Journal of Dentistry (n = 1), Dental Education (n = 1), Dental Research Journal (n = 1), Journal of Medical Education Development (n = 2), Horizons of Medical Education Development (n = 2)
Abstract presented at the national congress N (%)	11 (17)	**Congresses**: Annual Iranian Conference on Health Professions Education (n = 7), National Congress of Occupational Medicine (n = 2), Iranian Society of Medical Educationists (n = 2)
Grant N (%)	64 (100)	**Grant Sponsors**: Grant of University = 30 (46.9), National Grant of NASR***= 34 (53.1)
Award in educational festival N (%)	17 (27)	**Award**: ** Motahari Award in an Educational Festival in National Level = 7 (41.1), Motahari Educational Festival in University Level = 10 (58.8)
Promotion* N (%)	22 (34)	**Educational responsibility**: Dean of a school (n = 1), Vice-Chancellor of a school/educational hospital (n = 2), Director of an educational development office at school (n = 8), Director of the education department in schools (n = 2), Director of Education section of an educational department (n = 2), Member of the education committees in the vice-chancellor of the university (n = 7)
The number of projects with ongoing implementation over the two years following the ESP N (%)	52 (81)	
Conducting further SoTL projects after ESP N (%)	34 (53)	

*Defined as a new educational responsibility, appointment, or leadership role (e.g., education committee chair, rotation director, associate residency program director, and fellowship director).

** In the context, the annual SoTL festival that called Motahari, is held in the International Conference on Health Profession Education by the Ministry of healthcare and education at Iran. The program aimed to highlight, and exemplify best-practice in healthcare professions education, improving the education process and outcomes of other institutions around the Iranian context. Each year applications submit SoTL activities and their greatest achievements in a variety of SoTL domain. The SoTL activities assessed through the defined criteria of SoTL. The assessment was conducted in two stages in the levels of universities and national in the Ministry. In the studied context, Motahari awards is known as one of the output of SoTL training courses such as ESP.

***NASR: National Agency for Strategic Research in Medical Education

The budget of 64 SoTL projects reported in Table 4 was approximately 3000 million IRR (each applicant requested 30–50 million IRR). The results indicated that the change rate increased in the measures comprising the number of grants (966%), their budgets (2042.86% %), and the number of publications (1550%) from 2017 to 2022 compared to those published between 2011 and 2016. The increase rate means the percentage change in the value of measures positively growth over a period of time.

## Discussion

The ESP was implemented as a longitudinal FDP using project-based learning, mentoring, and collaborative learning principles. It was found that the evaluations of the ESP based on the Kirkpatrick model were favorable at all levels.

The reform of traditional educational strategies and methods in universities requires the improvement of educators’ capabilities to design, implement, and evaluate SoTL activities in educational communities [3]. Love and colleagues [45] demonstrated that ‘perceived rewards for change’ and ‘supportive work environment’ were the main factors in the success of FDPs. In the present study, the goals, obvious consequences (e.g., awards, publication, and promotion), and implicit consequences (e.g., improving teaching performance and improving the effectiveness of education) at the beginning of the ESP were explained to create a positive perception of change among the educator. Moreover, collaborative learning opportunities and mentoring were enforced in the training and project-based stages of the ESP to establish a supportive environment. Likewise, Chandran acknowledged that the mentoring and support approach through creating a network of peers and colleagues at the institution impacted the educators’ success in the ESP [3]. The present findings also revealed that effective and encouraging communication, useful participation, and increasing motivation were the main factors in mentoring from the educator’s perspective. Similarly, Clarke et al. [46] showed that support and mentoring in educational scholarship was a positive experience for their educators.

The three common components, i.e., interaction and collaborative activities, exchanging experiences, and reflection, were determined as the main factors leading to the positive reaction of the educators. Steinert and colleagues [1] acknowledged successful features of FDPs, including experiential learning, opportunities for feedback and reflection, longitudinal program design, and institutional support. They stated that the collaboration network facilitated the formation of a faculty development community and participation in joint activities, causing positive reactions from faculties.

In this study, a project-based learning approach was used in longitudinal programs as the main feature of ESP. Moreover, practical learning is recommended as a valuable opportunity for educators to experience the transfer of learning by applying theoretical knowledge into practice [1, 2, 22]. While implementing the SoTL project, educators experienced a SoTL cycle in different stages, such as setting goals, designing and implementing SoTL, critique reflection, and dissemination. The ESP provided circumstances of learning from peers and colleagues by critical reflective practice and dissemination in networks and meetings. Thus, the educators experienced an experiential learning process through learning in action, reflection, collaboration, and mentoring. These learning situations may influence positive outcomes in the levels of learning and behavior.

Similarly, OKeefe et al. revealed that the relationships among educators in faculty development programs assisted their learning [47]. In line with the present study, Chandran and colleagues [3] indicated that the FDP improved participants’ cooperation by focusing on educational scholarship. A study by Steinert and colleagues [1] consistently indicated that educators preferred longitudinal faculty development for experiential learning and group working. They showed that educators were satisfied with the practical learning experiences in these activities. Using diverse methods in the experiential learning process led to a high level of participants’ satisfaction.

The current results showed that the change rate of the outputs increased significantly compared to those in the previous period. The ESP influenced these results by practical learning situations, mentoring, and creating cooperation networks. This ESP encouraged the educators to engage in SoTL projects with obvious and implicit consequences. The beneficial opportunities significantly increased the change rate of the outputs. These positively impacted the output growth rate in the results level compared to the previous period when only grants were given to projects. A previous qualitative study showed that individual motivational factors affected educators’ success in implementing SoTL projects. Developing infrastructures and system support were also recommended to achieve the goals of ESP [12].

The current results showed that the ESP’s outputs, including projects with ongoing implementation over the two years following the ESP, the acquisition of grants, and the conduction of further SoTL projects after ESP, were higher than other outputs. These results indicated that educators have been able to learn and apply critical skills to change education using SoTL activities. Likewise, Macario and colleagues [16] indicated that educational products, including promotion and the conduction of the SoTL project, were the highest outcomes of the ESP at Stanford University. Love and colleagues [45](2019) also illustrated that the Flexible Development Program (FDP) within the Education Research domain resulted in 95% of educators successfully applying the acquired skills. It should be mentioned that utilizing practical learning may affect the results. Chan [48]et al. 48 displayed that articles and books were the most achievements of longitudinal FDP. They stated that this achievement was valuable because it shows that the FDP was useful for promoting educational scholarship in their context.

### Lessons learned

Supportive mechanisms such as mentoring and networking were important in preparing for longitudinal involvement in the SoTL project. These mechanisms positively impacted educators’ learning and implementation of SoTL as an educational change project in the educational community. Creating a structured process focusing on regular planning and support mechanisms positively impacted learner-related outcomes.

### Limitations

This study was conducted in one university, which limits the generalizability of the results. The non-randomization and lack of a control group to control biases due to the voluntary participation of the educators were also among the limitations of the present study. Additionally, self-reported data, the absence of baseline data, and the potential for inconsistency in the assessment/judgment of SoTL projects were some restrictions of this study. The evaluation duration of the outcomes of the initial cohorts was completed, but in the case of cohorts 4 and 5, the results may change over time.

## Conclusion

As an institute-based longitudinal program, the ESP enhanced the learner-related outcomes (in four levels of reaction, learning, behavior, and results). The current results demonstrated that the change rate of the outputs increased significantly compared to those in the previous period. Creating practical learning and supportive mechanisms influenced the results. So, it is recommended to conduct studies on evaluating the impact of the ESP on the development of scholarly culture in the educational community and also the explanation of the hidden curriculum of ESP in future studies. Moreover, it is suggested to assess the impact of the ESP on the different roles of educators in medical science systems, including educational leaders, curriculum developers, and evaluators.

### Electronic supplementary material

Below is the link to the electronic supplementary material.

Supplementary Material 1

## Data Availability

The datasets used and/or analyzed during the current study are available from the corresponding author upon reasonable request.

## References

[1] Steinert Y, Mann K, Anderson B, Barnett BM, Centeno A, Naismith L (2016). A systematic review of faculty development initiatives designed to enhance teaching effectiveness: a 10-year update: BEME Guide No. 40. Med Teach.

[2] Steinert Y, Mann K, Centeno A, Dolmans D, Spencer J, Gelula M (2006). A systematic review of faculty development initiatives designed to improve teaching effectiveness in medical education: BEME Guide No. 8. Med Teach.

[3] Chandran L, Gusic ME, Lane JL, Baldwin CD (2017). Designing a national longitudinal faculty development curriculum focused on educational scholarship: process, outcomes, and lessons learned. Teach Learn Med.

[4] Jolly B. Faculty development for organizational change. Faculty development in the health professions: a focus on research and practice. Springer; 2013. pp. 119–37.

[5] McLean M, Cilliers F, Van Wyk JM (2008). Faculty development: yesterday, today and tomorrow. Med Teach.

[6] Wilkerson L, Irby DM (1998). Strategies for improving teaching practices: a comprehensive approach to faculty development. Acad Medicine: J Association Am Med Colleges.

[7] Harden RM, Laidlaw JM. Essential skills for a medical teacher: an introduction to teaching and learning in medicine. Elsevier Health Sciences; 2020.

[8] Raff BS, Arnold J (2001). Faculty Development: an approach to scholarship. Nurse Educ.

[9] Pelger S, Larsson MJ (2018). Advancement towards the scholarship of teaching and learning through the writing of teaching portfolios. Int J Acad Dev.

[10] Laksov KB (2020). The pedagogical ambassadorship programme as an approach to academic development. Högre Utbildning.

[11] Irby DM, O’sullivan PS (2018). Developing and rewarding teachers as educators and scholars: remarkable progress and daunting challenges. Med Educ.

[12] Keshmiri F (2023). A developmental pathway toward leadership for educational change: the educators’ experiences of the educational scholar program. BMC Med Educ.

[13] Kordestani Moghaddam A, Mirzazadeh AJ (2019). Medical Education Scholars Program: an approach to development of scholars in education in Tehran University of Medical Sciences. Adv Med Educ Pract.

[14] Steinert Y (2017). Faculty development: from program design and implementation to scholarship. GMS J Med Educ.

[15] Lawrence DJ (2010). A teaching scholar program in chiropractic education. J Can Chiropr Assoc.

[16] Macario A, Tanaka PP, Landy JS, Clark SM, Pearl RG (2013). The stanford anesthesia faculty teaching scholars program: summary of faculty development, projects, and outcomes. J Graduate Med Educ.

[17] Muller JH, Irby DM (2006). Developing educational leaders: the teaching scholars program at the University of California, San Francisco, School of Medicine. Acad Medicine: J Association Am Med Colleges.

[18] Moses AS, Skinner DH, Hicks E, O’Sullivan PS (2009). Developing an educator network: the effect of a teaching scholars program in the health professions on networking and productivity. Teach Learn Med.

[19] Thomas JW. A review of research on project-based learning. California; 2000.

[20] Kokotsaki D, Menzies V, Wiggins A (2016). Project-based learning: a review of the literature. Improving Schools.

[21] Rowland SL, Myatt PM (2014). Getting started in the scholarship of teaching and learning: a how to guide for science academics. Biochem Mol Biol Educ.

[22] Steinert Y, Naismith L, Mann K (2012). Faculty development initiatives designed to promote leadership in medical education. A BEME systematic review: BEME Guide No. 19. Med Teach.

[23] Keshmiri F, Gandomkar R, Hejri SM, Mohammadi E, Mirzazadeh AJMT. Developing a competency framework for health professions education at doctoral level: the first step toward a competency based education. 2019;41(11):1298–306. 10.1080/0142159X.2019.1636952 31329019

[24] Zepeda SJ, Parylo O, Bengtson E (2014). Analyzing principal professional development practices through the lens of adult learning theory. Prof Dev Educ.

[25] Hämäläinen R, Vähäsantanen KJ (2011). Theoretical and pedagogical perspectives on orchestrating creativity and collaborative learning. Educational Res Rev.

[26] Billett SJ (2016). Learning through health care work: premises, contributions and practices. Med Educ.

[27] Haraldseid-Driftland C, Aase K, Wiig S, Billett SJ (2021). Developing a collaborative learning framework for resilience in healthcare: a study protocol. BMJ open.

[28] Plass JL, O’Keefe PA, Homer BD, Case J, Hayward EO, Stein M (2013). The impact of individual, competitive, and collaborative mathematics game play on learning, performance, and motivation. J Educ Psychol.

[29] Nelson JK, Hjalmarson M, editors. Faculty development groups for interactive teaching. 2015 ASEE Annual Conference & Exposition; 2015.

[30] Taylor DC, Hamdy H (2013). Adult learning theories: implications for learning and teaching in medical education: AMEE Guide No. 83. Med Teach.

[31] Steinert Y, Mann KV (2006). Faculty development: principles and practices. J Vet Med Educ.

[32] Smidt A, Balandin S, Sigafoos J, Reed VAJJ (2009). The Kirkpatrick model: a useful tool for evaluating training outcomes. J Intellect Dev Disabil.

[33] Frye AW, Hemmer PA (2012). Program evaluation models and related theories: AMEE guide no. 67. Med Teach.

[34] Bates R (2004). A critical analysis of evaluation practice: the Kirkpatrick model and the principle of beneficence. Evaluation Program Planning.

[35] Cahapay MJ (2021). Kirkpatrick model: its limitations as used in higher education evaluation. Int J Assess Tools Educ.

[36] Glassick C (2000). Boyer’s expanded definitions of scholarship, the standards of assessing scholarship, and the elusiveness of the scholarship of teaching. Acad Med.

[37] Thurston A, Topping KJ, Tolmie A, Christie D, Karagiannidou E, Murray PJ (2010). Cooperative Learning in Science: follow-up from primary to high school. Int J Sci Educ.

[38] Thistlethwaite JE, Davies D, Ekeocha S, Kidd JM, MacDougall C, Matthews P (2012). The effectiveness of case-based learning in health professional education. A BEME systematic review: BEME Guide No. 23. Med Teach.

[39] AhmariTehran H, Mohammadimehr M, Keshmiri F (2022). A practical guide for conducting scholarship of teaching and learning (SoTL): an approach to developing the innovative educational process. Strides Dev Med Educ.

[40] Elo S (2007). Kynga¨ s H. The qualitative content analysis process. Res Methodol.

[41] Glassick CE (2000). Reconsidering scholarship. J Public Health Manage Practice: JPHMP.

[42] Keshmiri F. The effect of blended learning approaches in faculty development programs. Strides in Development of Medical Education. 2022;19(1).

[43] Zarrabi M, Imanieh M, Zarrabi K, Masjedi M, Kojuri J, Amini M (2017). Designing and organizing mentoring at shiraz medical school and reinforcing deep knowledge–based education using mentoring. J Med Cultivation.

[44] Cohen-Schotanus J, van der Vleuten CP (2010). A standard setting method with the best performing students as point of reference: practical and affordable. Med Teach.

[45] Love JN, Yarris LM, Santen SA, Kuhn GJ, Gruppen LD, Coates WC (2016). A novel specialty-specific, collaborative faculty development opportunity in education research: program evaluation at five years. Acad Med.

[46] Clarke SO, Jordan J, Yarris LM, Fowlkes E, Kurth J, Runde D (2018). The view from the top: academic emergency department chairs’ perspectives on education scholarship. AEM Educ Train.

[47] O’Keefe M, Lecouteur A, Miller J, McGowan U (2009). The Colleague Development Program: a multi-disciplinary program of peer observation partnerships. Med Teach.

[48] Chan TM, Gottlieb M, Sherbino J, Cooney R, Boysen-Osborn M, Swaminathan A (2018). The ALiEM faculty incubator: a novel online approach to faculty development in education scholarship. Acad Medicine: J Association Am Med Colleges.

