# Supplementary material for: The effect of the Educational Scholar Program as a longitudinal faculty development program on the capability of educators as scholars
Source: BMC Med Educ. 2023 Sep 22;23:691. doi: 10.1186/s12909-023-04682-7 (PMC10517549; doi:10.1186/s12909-023-04682-7)
Supplement: Supplementary file 1 — Supplementary Material 1 [file 12909_2023_4682_MOESM1_ESM.docx]

**Appendices:**

**Appendix 1:**

The JIGSAW, an interactive method, was implemented in four steps: 1) a topic for study was assigned for each educator, 2) educators participated in non-homogenous small groups and studied their topics, 3) they contributed to a homogenous group according to their topics and actively participated to learn, finally, 4) they came back to the non-homogenous group and shared their topics with others. Therefore, each topic was then repeated and reviewed for each person several times (1).

Reference

1. Thurston A, Topping KJ, Tolmie A, Christie D, Karagiannidou E, Murray PJ. Cooperative Learning in Science: Follow‐up from primary to high school. International Journal of Science Education. 2010;32(4):501-22.
